# Supplementary material for: Prognostic genome and transcriptome signatures in colorectal cancers
Source: Nature. 2024 Aug 7;633(8028):137–46. doi: 10.1038/s41586-024-07769-3 (PMC11374687; doi:10.1038/s41586-024-07769-3)
Supplement: Supplementary file 1 — Supplementary Figs. 1–12, Supplementary Tables 5, 6 and 17 and the captions for Supplementary Tables 1–30. [file 41586_2024_7769_MOESM1_ESM.docx]

**Prognostic genome and transcriptome signatures in colorectal cancers**

Luís Nunes^1,*,†^, Fuqiang Li^2,3,4,*^, Meizhen Wu^2,3,4,*^, Tian Luo^2,3,4,*^, Klara Hammarström^1^, Emma Torell^1^, Ingrid Ljuslinder^5^, Artur Mezheyeuski^1^, Per-Henrik Edqvist^1^, Anna Löfgren-Burström^6^, Carl Zingmark^6^, Sofia Edin^6^, Chatarina Larsson^1^, Lucy Mathot^1^, Erik Osterman^1^, Emerik Osterlund^1^, Viktor Ljungström^1^, Inês Neves^1^, Nicole Yacoub^1^, Unnur Guðnadóttir^1^, Helgi Birgisson^7^, Malin Enblad^7^, Fredrik Ponten^1^, Richard Palmqvist^6^, Xun Xu^2^, Mathias Uhlén^8,9^, Kui Wu^2,3,4,**^, Bengt Glimelius^1,**^, Cong Lin^2,3,4,**^, Tobias Sjöblom^1,**^

^1^ Department of Immunology, Genetics and Pathology, Science for Life Laboratory, Uppsala University, Uppsala, Sweden.

^2^ HIM-BGI Omics Center, Hangzhou Institute of Medicine (HIM), Chinese Academy of Sciences (CAS), BGI Research, Hangzhou 310000, China

^3^ Guangdong Provincial Key Laboratory of Human Disease Genomics, Shenzhen Key Laboratory of Genomics, BGI Research, Shenzhen 518083, China

^4^ Institute of Intelligent Medical Research (IIMR), BGI Genomics, Shenzhen 518083, China

^5^ Department of Radiation Sciences, Oncology, Umeå University, Umeå, Sweden

^6^ Department of Medical Biosciences, Pathology, Umeå University, Umeå, Sweden

^7^ Department of Surgical Sciences, Uppsala University, Akademiska sjukhuset, Uppsala, Sweden.

^8^ Department of Neuroscience, Karolinska Institutet, Stockholm, Sweden

^9^ Science for Life Laboratory, Department of Protein Science, KTH-Royal Institute of Technology, Stockholm, Sweden

^*^ These authors contributed equally to this work: Luís Nunes, Fuqiang Li, Meizhen Wu and Tian Luo

^**^ These authors jointly supervised this work: Kui Wu, Bengt Glimelius, Cong Lin and Tobias Sjöblom. e-mail: wukui@genomics.cn; bengt.glimelius@igp.uu.se; lincong@genomics.cn; tobias.sjoblom@igp.uu.se **(lead contact)**

† Present address: Department of Molecular Oncology, Institute for Cancer Research, Oslo University Hospital, Oslo, Norway

**Supplementary Information Guide**

**Supplementary Figures:**

Supplementary Figure 1………………………………………………………………..3

Supplementary Figure 2………………………………………………………………..5

Supplementary Figure 3………………………………………………………………..7

Supplementary Figure 4………………………………………………………………..8

Supplementary Figure 5………………………………………………………………10

Supplementary Figure 6………………………………………………………………12

Supplementary Figure 7………………………………………………………………13

Supplementary Figure 8………………………………………………………………14

Supplementary Figure 9………………………………………………………………15

Supplementary Figure 10..……………………………………………………………17

Supplementary Figure 11………..……………………………………………………19

Supplementary Figure 12..……………………………………………………………20

**Supplementary Tables (non-excel tables):**

Supplementary Table 5………….……………………………………………………22

Supplementary Table 6..………………….…………………………………………..23

Supplementary Table 17……..……………….………………………………..……..24

**Supplementary Tables Captions:**

Supplementary Tables 1-30 Captions..…………...…………………………………..25

**
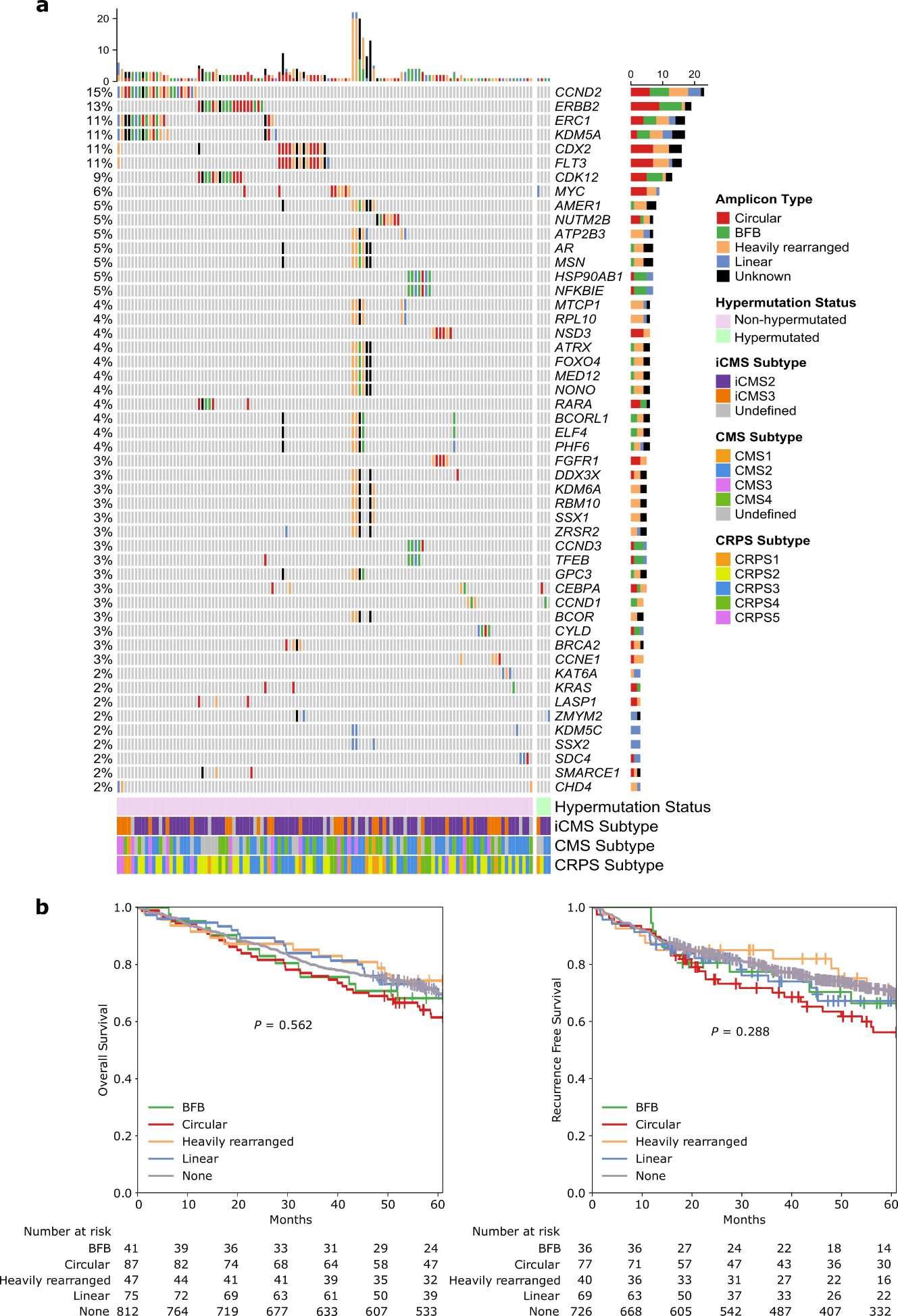
Supplementary Figures:**

**Supplementary Figure 1. Extrachromosomal DNA landscape of colorectal cancer.** Tumours with extrachromosomal DNA (ecDNA) harbouring oncogenes were analysed. **a**, Oncoplot of the top 50 oncogenes observed with ecDNA amplicons in non-hypermutated

**Supplementary Figure 1 (continued).** (left columns) and hypermutated (last 4 right columns) tumours. The fraction of tumours with ecDNA involving the oncogene is shown to the left. Number of ecDNA amplicons are shown by type for each tumour (top) and for each oncogene (right). **b**, Overall and recurrence free survival Kaplan-Meier curves for samples classified to be dominated by different types of ecDNAs. The tumours were classified based on which amplicons were present in the sample as previously described (<https://doi.org/10.1038/s41588-020-0678-2>). Log-rank test with *P*<0.05 was considered significant. BFB, breakage-fusion-break.


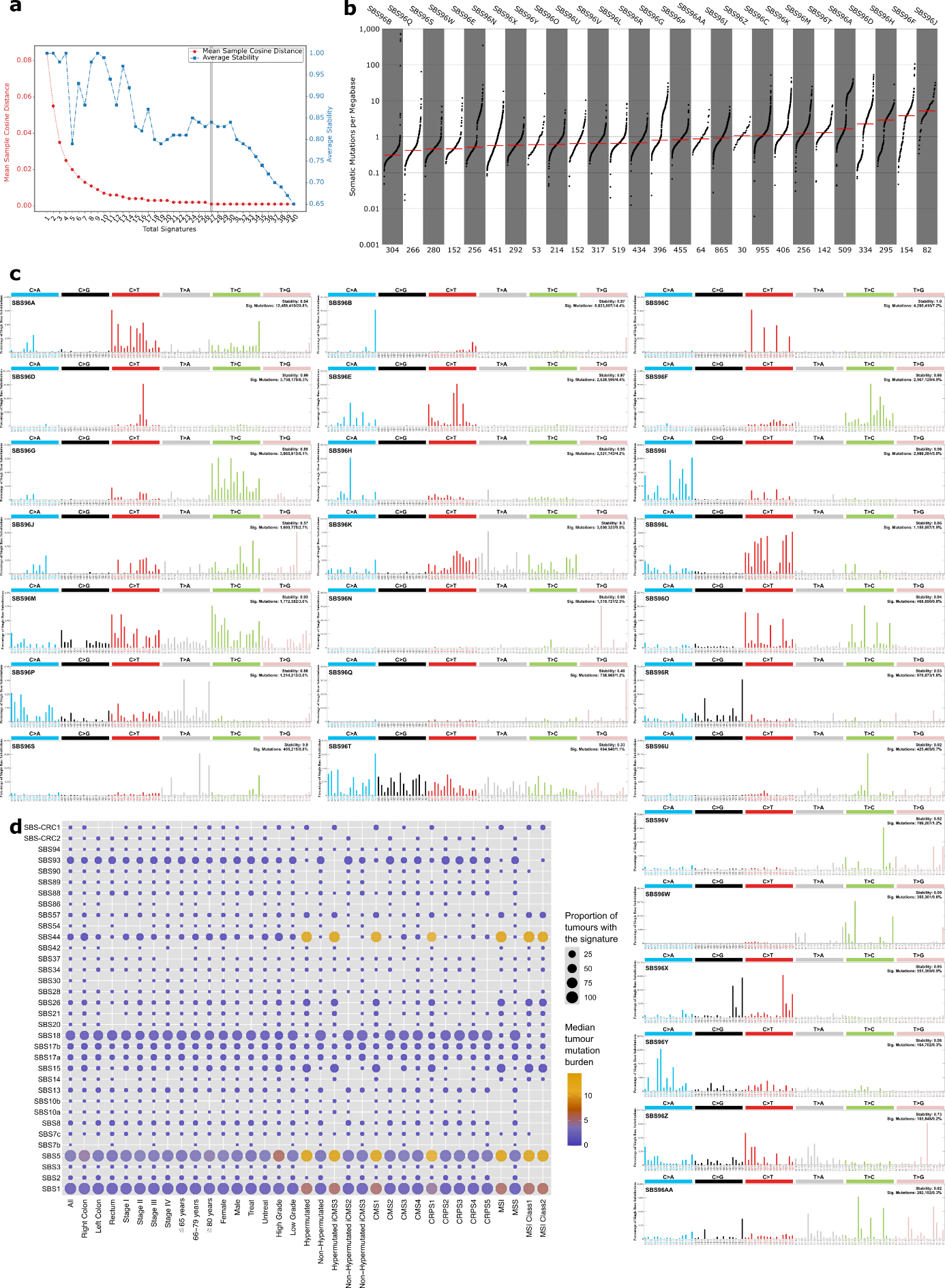


**Supplementary Figure 2. *De novo* extraction of single-base substitution (SBS) mutational signatures.** **a**, Hierarchical *de novo* extraction of SBS signatures from all tumours and estimation of the optimal solution (number of signatures marked with the grey line) based on the stability and accuracy of all 40 solutions. **b**, Mutation burden per megabase of each somatic SBS signature sorted by median (red line), with each dot representing one tumour and the

**Supplementary Figure 2 (continued).** number of tumours with signature indicated at the bottom. **c**, Profiles of the 27 SBS mutational signatures. **d**, Decomposed mutational signature landscape showing known and novel (top two rows) SBS signatures in relation to clinical and molecular characteristics. The circle size shows proportion of tumours with the signature and colour shows the median tumour mutation burden (TMB) according to the keys on the right side.


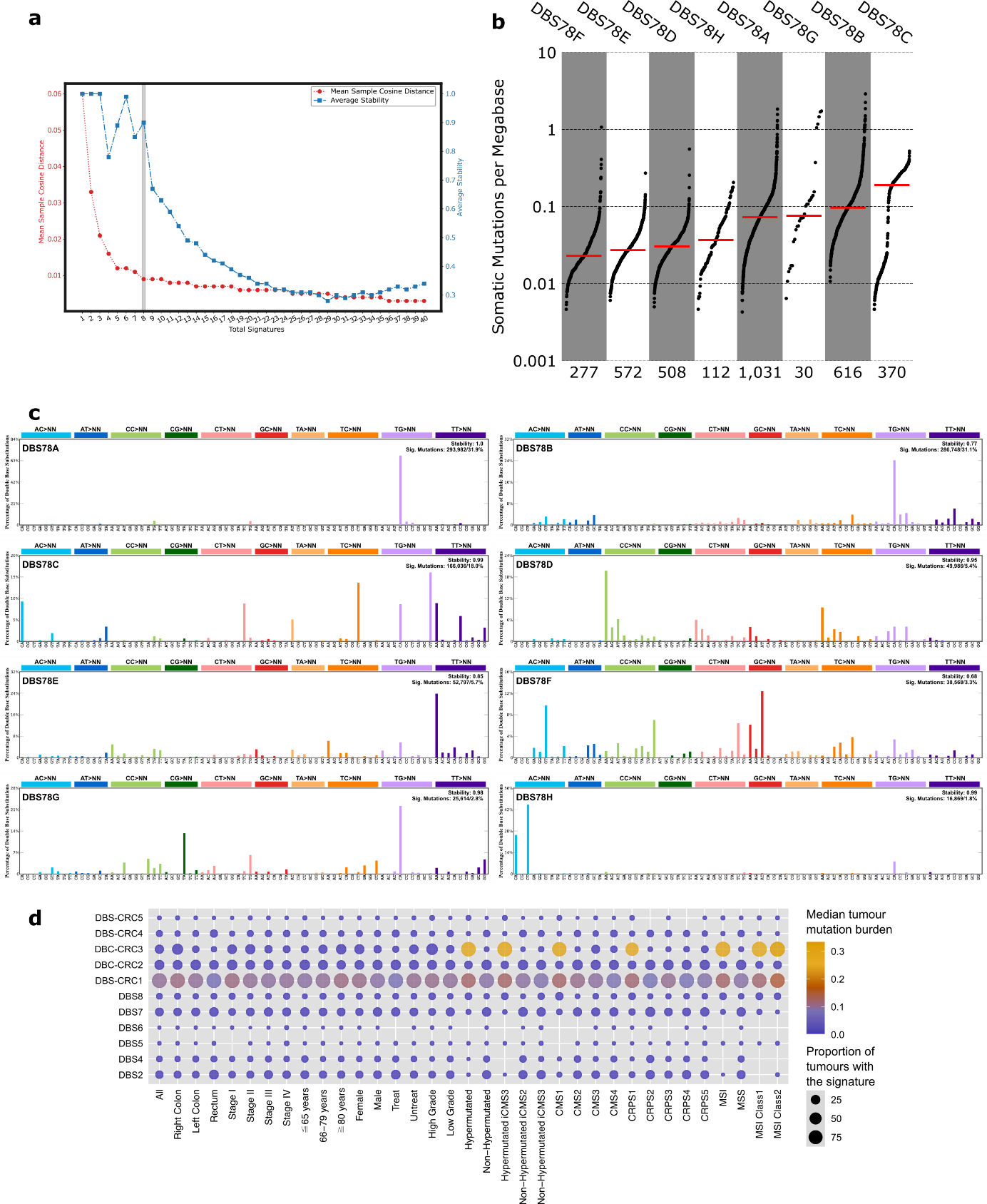


**Supplementary Figure 3. *De novo* extraction of doublet-base substitution (DBS) mutational signatures**. **a**, Hierarchical *de novo* extraction of DBS signatures from all tumours and estimation of the optimal solution (number of signatures marked with the grey line) based on the stability and accuracy of all 40 solutions. **b**, Mutation burden per megabase of each somatic DBS signature sorted by median (red line), with each dot representing one tumour and the number of tumours with signature indicated at the bottom. **c**, Profiles of the 8 DBS mutational signatures. **d**, Decomposed mutational signature landscape showing known and novel (top five rows) DBS signatures in relation to clinical and molecular characteristics. The circle size shows the proportion of tumours with the signature and colour show the median tumour mutation burden (TMB) according to the keys on the right side.


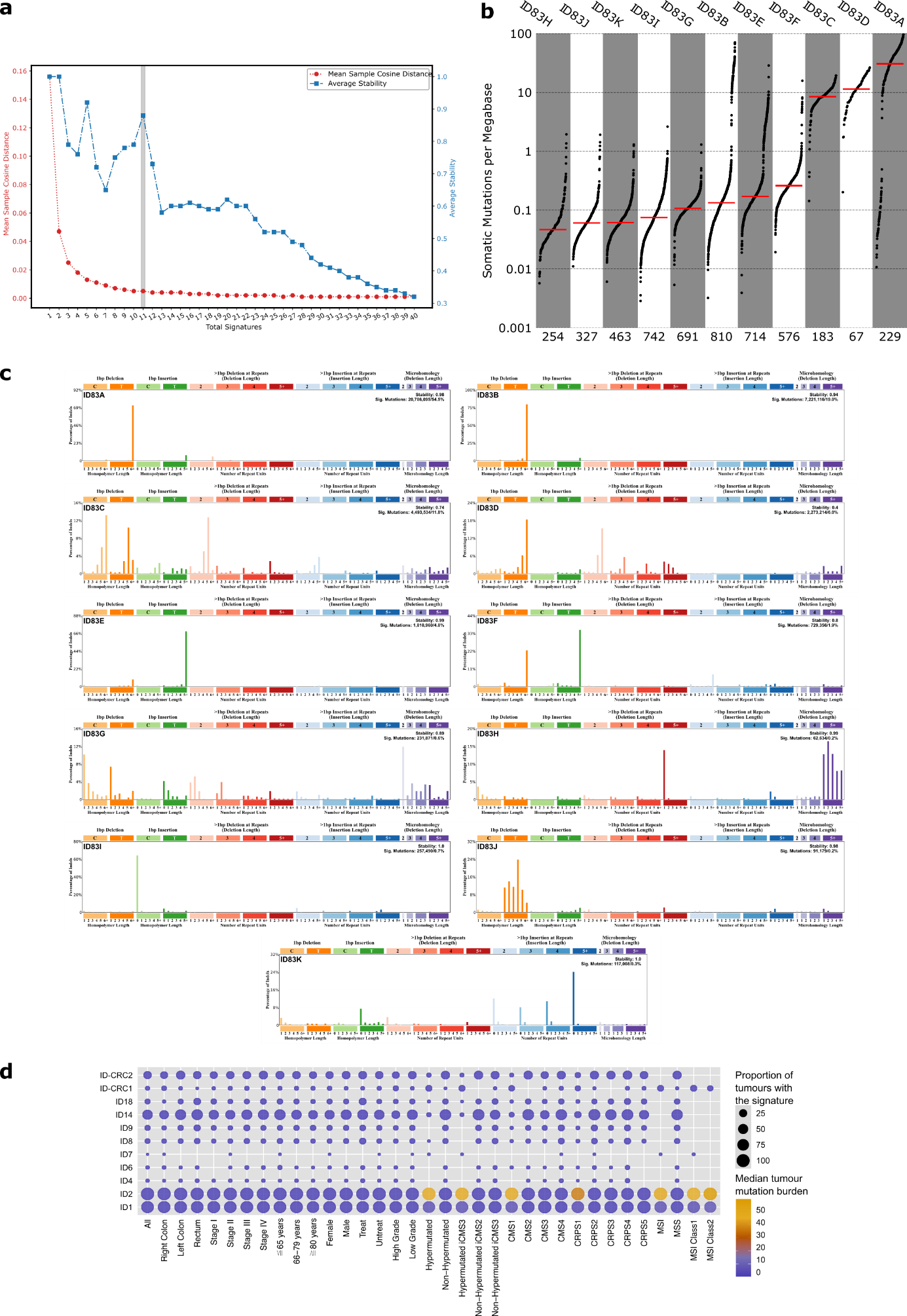


**Supplementary Figure 4. *De novo* extraction of small insertion and deletion (ID) mutational signatures**. **a**, Hierarchical *de novo* extraction of ID signatures from all samples and estimation of the optimal solution (number of signatures marked with the grey line)

**Supplementary Figure 4.** based on the stability and accuracy of all 40 solutions. **b**, Mutation burden per megabase of each somatic ID signature sorted by median (red line) with each dot representing one tumour and the number of tumours with signature indicated at the bottom. **c**, Profiles of the 11 ID mutational signatures. **d**, Decomposed mutational signature landscape showing known and novel (top two) ID signatures in relation to clinical and molecular characteristics. The circle size shows proportion of tumours with the signature and colour shows the median tumour mutation burden (TMB) according to the keys on the right side.


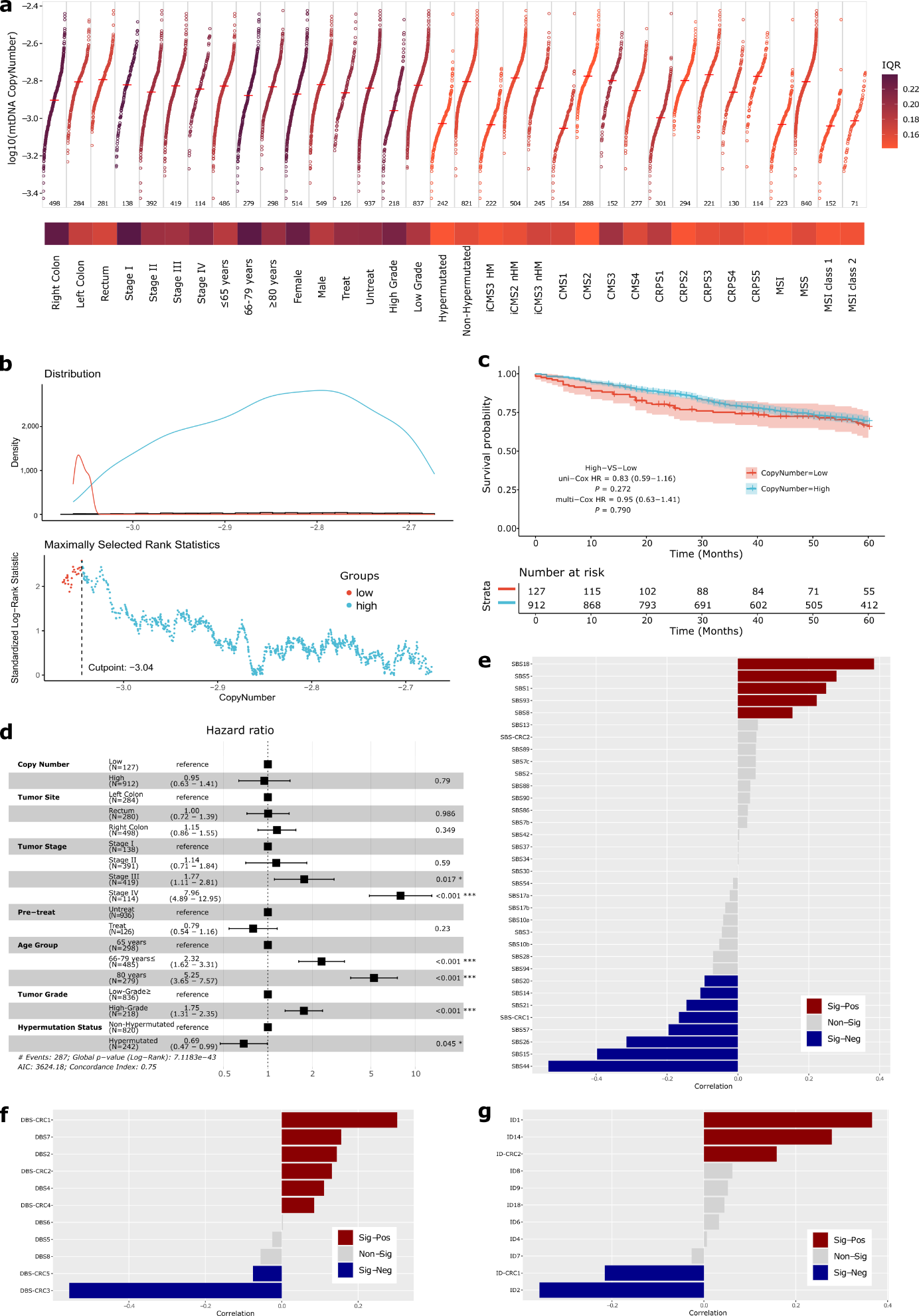


**Supplementary Figure 5. Mitochondrial copy number in 1,063 colorectal cancers. a**, Mitochondrial genome copy number (mtDNA-CN) landscape. For each feature, mtDNA-CN

**Supplementary Figure 5 (continued).** median was marked with a horizontal red line and variability was measured and coloured according to the interquartile range (IQR). Each dot represents a tumour and number of samples included in each feature is shown at the bottom. **b**, A cut point (dashed line) was defined for low (red) and high (blue) mtDNA-CN according to their ranked density distribution. **c**, Overall survival for mtDNA-CN groups was calculated using Kaplan-Meier curves, with respective confidence intervals (shading) and number of patients at risk (bottom). **d**, Overall survival was validated with multivariable Cox regression with multiple factors, with hazard ratios represented as square boxes and confidence intervals as ranges. **e-g**, Spearman correlation analyses between mtDNA-CN and single-base substitution (SBS; **e**), doublet-base substitution (DBS; **f**), and small insertions and deletions (ID; **g**) mutational signatures. Positive (red), non-significant (grey) and negative (blue) correlations defined by Spearman’ rank correlation and FDR<0.05.


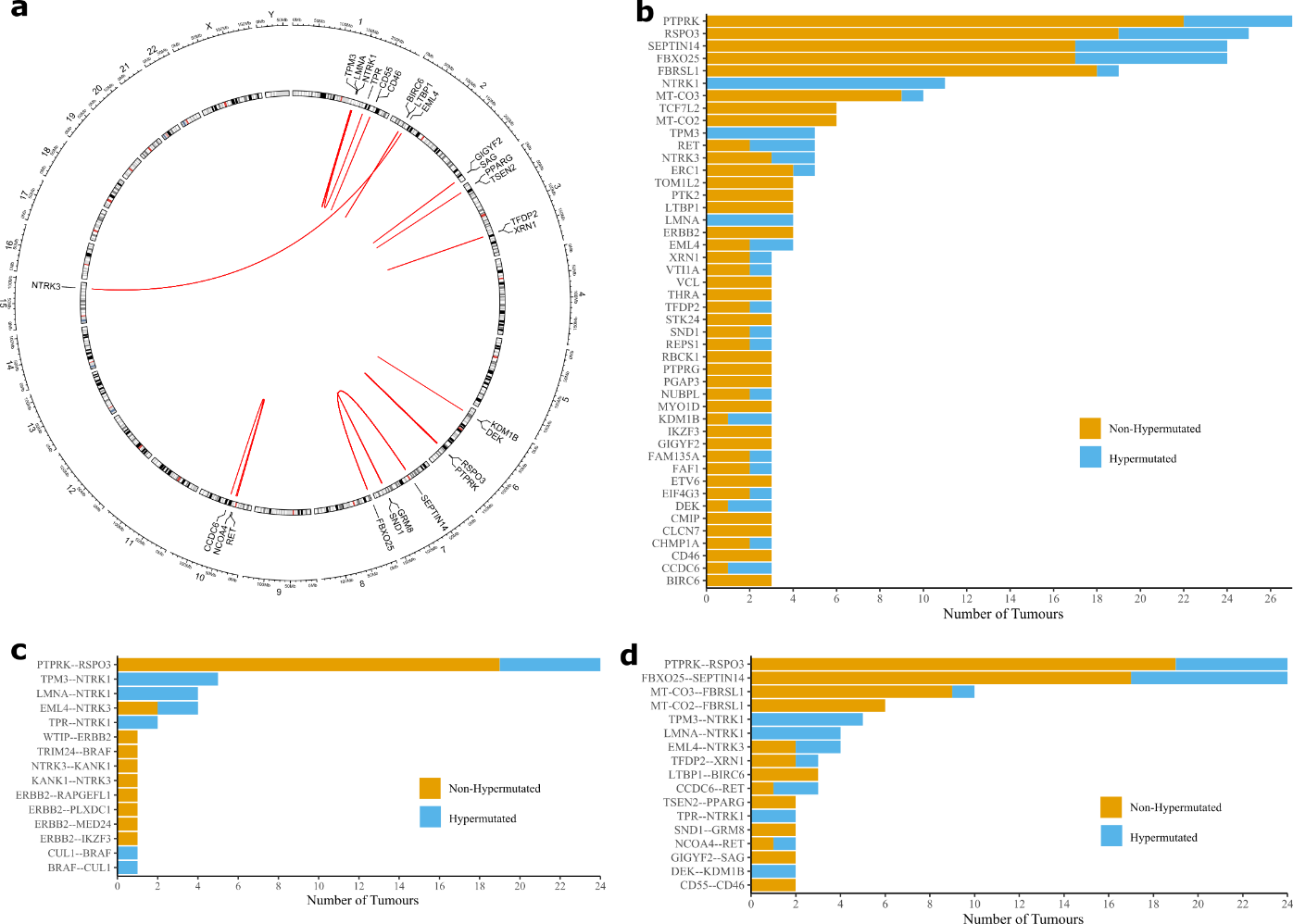


**Supplementary Figure 6. In-frame gene fusions detected by expression analysis.** Fusion genes were detected in RNAseq data from the 1,063 colorectal tumours using STAR-Fusion and Arriba. **a**, Circos plot of recurrent fusions displayed by chromosome location, excluding mitochondrial DNA fusions. **b**, Top 50 genes recurrently included in fusions displayed by sample hypermutation status. **c**, Recurrent colorectal cancer fusions described by Filippo *et al.* (<https://doi.org/10.3390/ijms20215319>) observed in this cohort, with indication of sample hypermutation status. **d**, Top 20 recurrent expressed gene fusions, with indication of hypermutation status of affected samples.


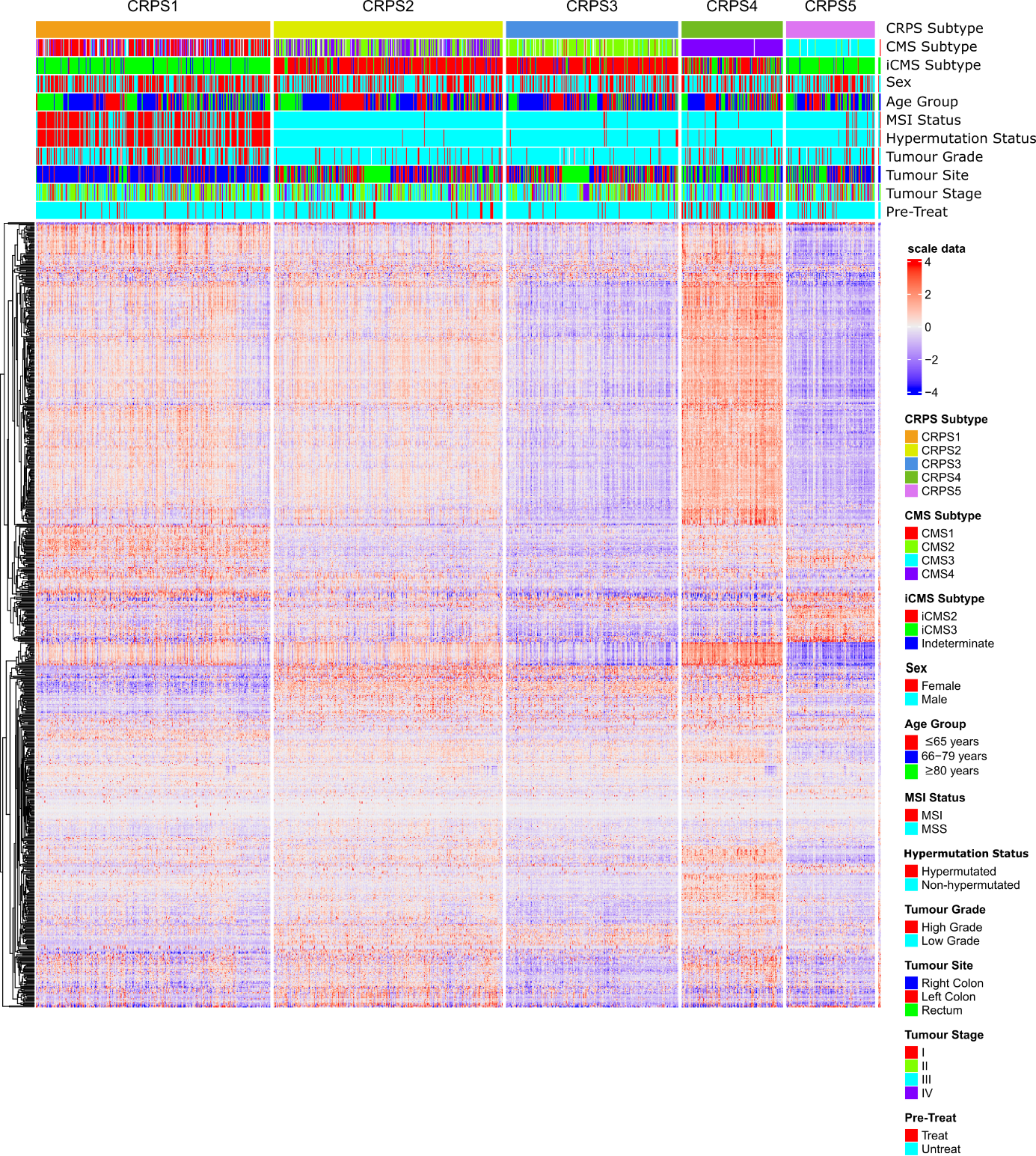


**Supplementary Figure 7. Colorectal cancer prognostic subtypes (CRPS) marker genes expression.** A heatmap displaying the marker genes in each CRPS subtype, where red indicates high expression and blue low expression of a particular gene. Other clinical and genomic features represented on top of the heatmap according to respective colour keys. The subtypes were identified by unsupervised clustering of transcriptomes from 1,063 CRCs using Seurat (version 4.1.0). Potential batch effects or source differences between samples were corrected by Celligner. The stability of the clusters was assessed by Jaccard similarity index and the preferred clustering result (resolution=0.9, PC=20, K=20) was determined by scclusteval. The Intrinsic CMS (iCMS) subtype classification was performed as previously described (<https://doi.org/10.1038/s41588-022-01100-4>) and cases were defined as indeterminate if permutation-based FDR≥0.05.


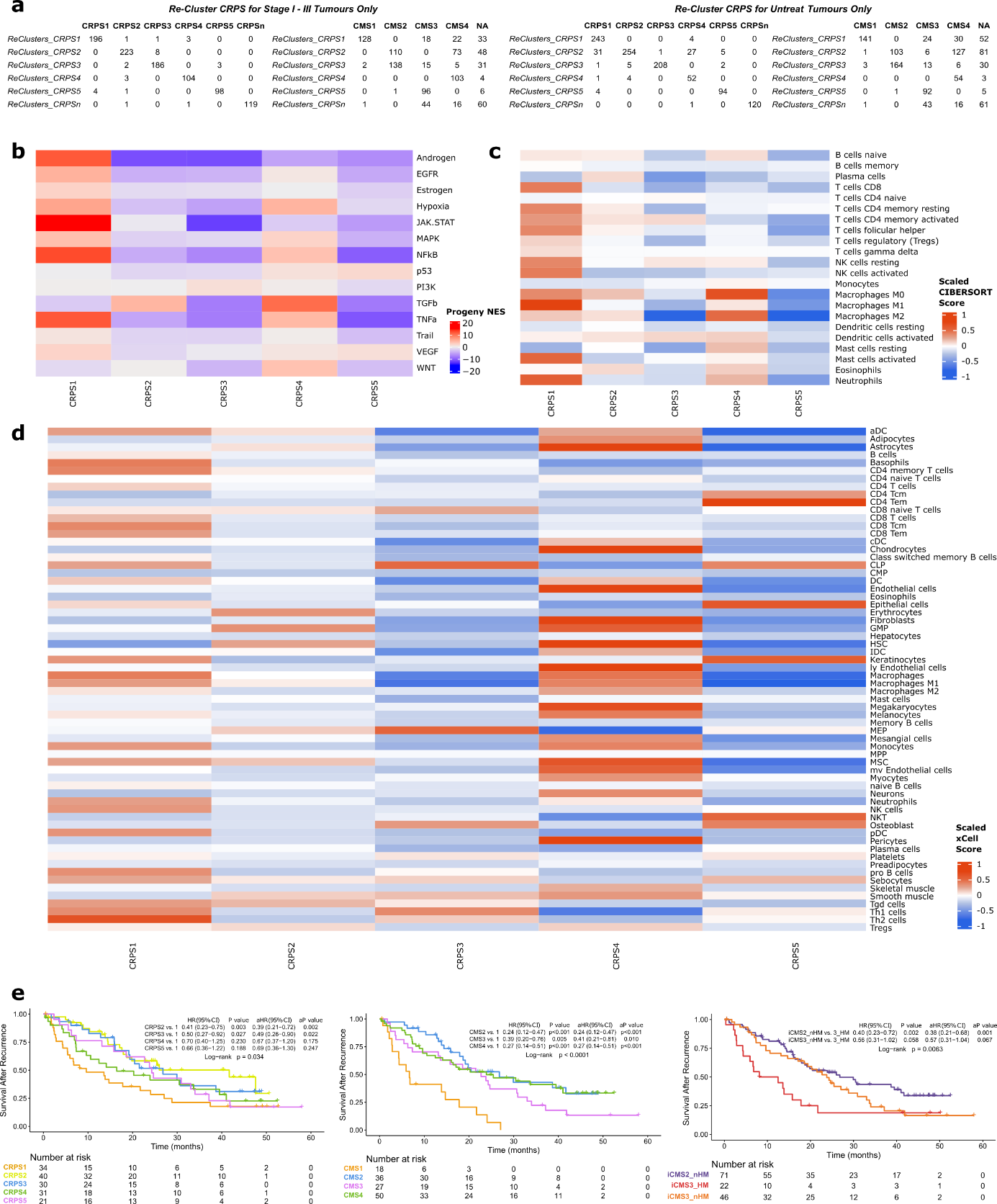


**Supplementary Figure 8. CRPS subtyping is robust to removal of stage IV or pre-treated tumours**. **a**, Distribution of cases from re-clustering by CRPS (first column) when stage IV or pre-treated tumours were excluded. This leads to similar CRPS and CMS classification (head row) as the original clustering where all cases, untreated and pre-treated at stages I-IV were included. CRPS after removal of pre-treated tumours revealed similar Progeny gene pathway enrichment (**b**) and predicted immune cell composition by CIBERSORT (**c**) and xCell (**d**). **e**, Survival after recurrence shown by CRPS, CMS and iCMS subgroups calculated with Kaplan-Meier curves and multivariable cox with or without adjustment for tumour stage, and log-rank test.


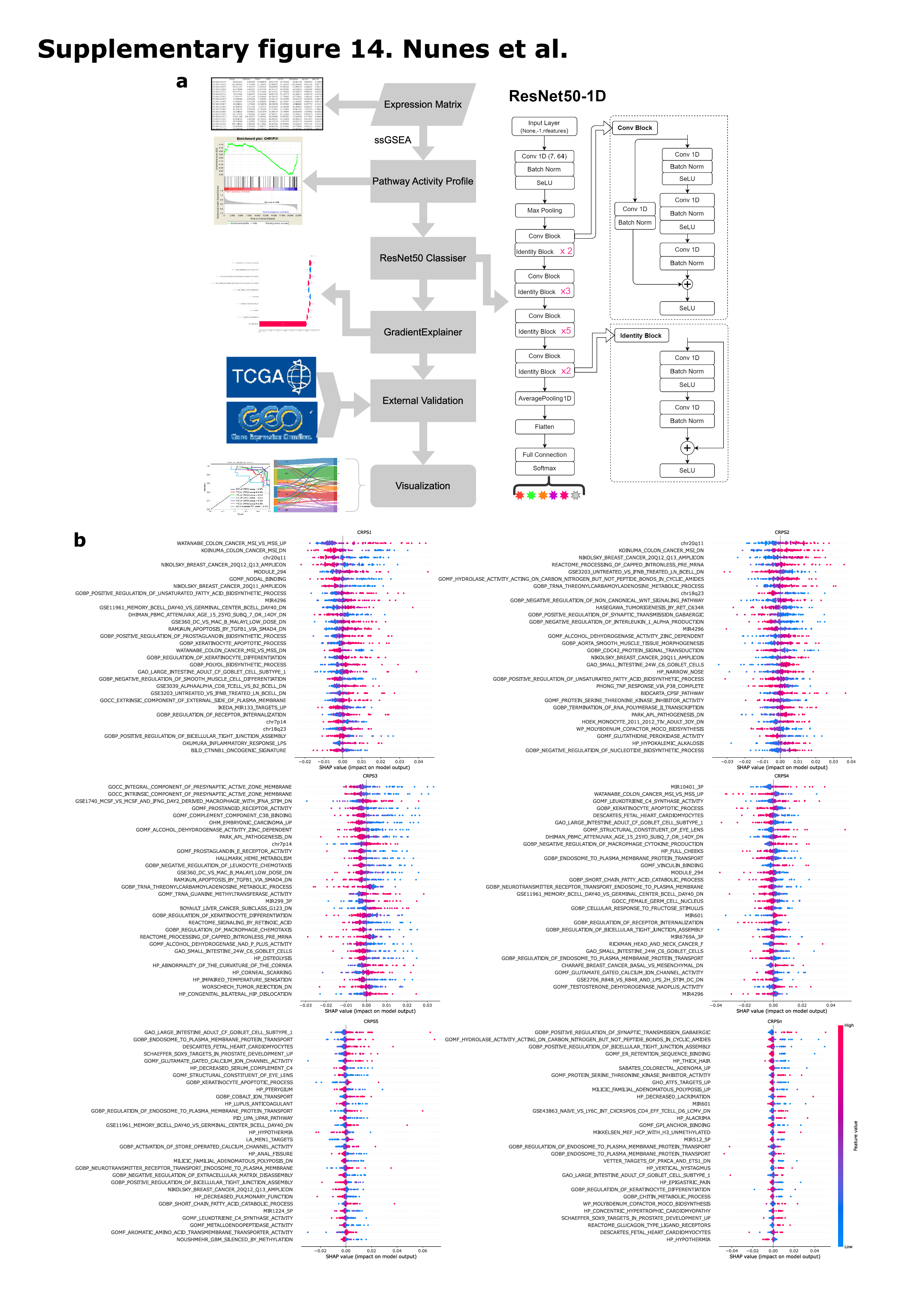


**Supplementary Figure 9. Deep learning model for CRPS classification and model**

**Supplementary Figure 9 (continued). interpretation at the pathway level. a**, A deep neural network architecture for the CRPS classifier was built with gene expression data from the 1,063 colorectal cancers based on the ResNet50-1D deep residual learning framework. The input to the model was an 1D vector, which represents each gene set row of a sample (gs_1_, gs_2_, ..., gs_n_). This 1D vector was converted to a 2D matrix (1, n_features_) with np.reshape(), and the selected pathways were fit to resnet50-1D by Conv1D(). See Methods for more detailed information. **b**, Shapley Additive exPlanations (SHAP) was applied to CRPS classifications to explain model predictions. SHAP values are shown for the features that contributed significantly for each CRPS subtype and coloured by feature value. CRPSn, CRPS normal sample subtype.


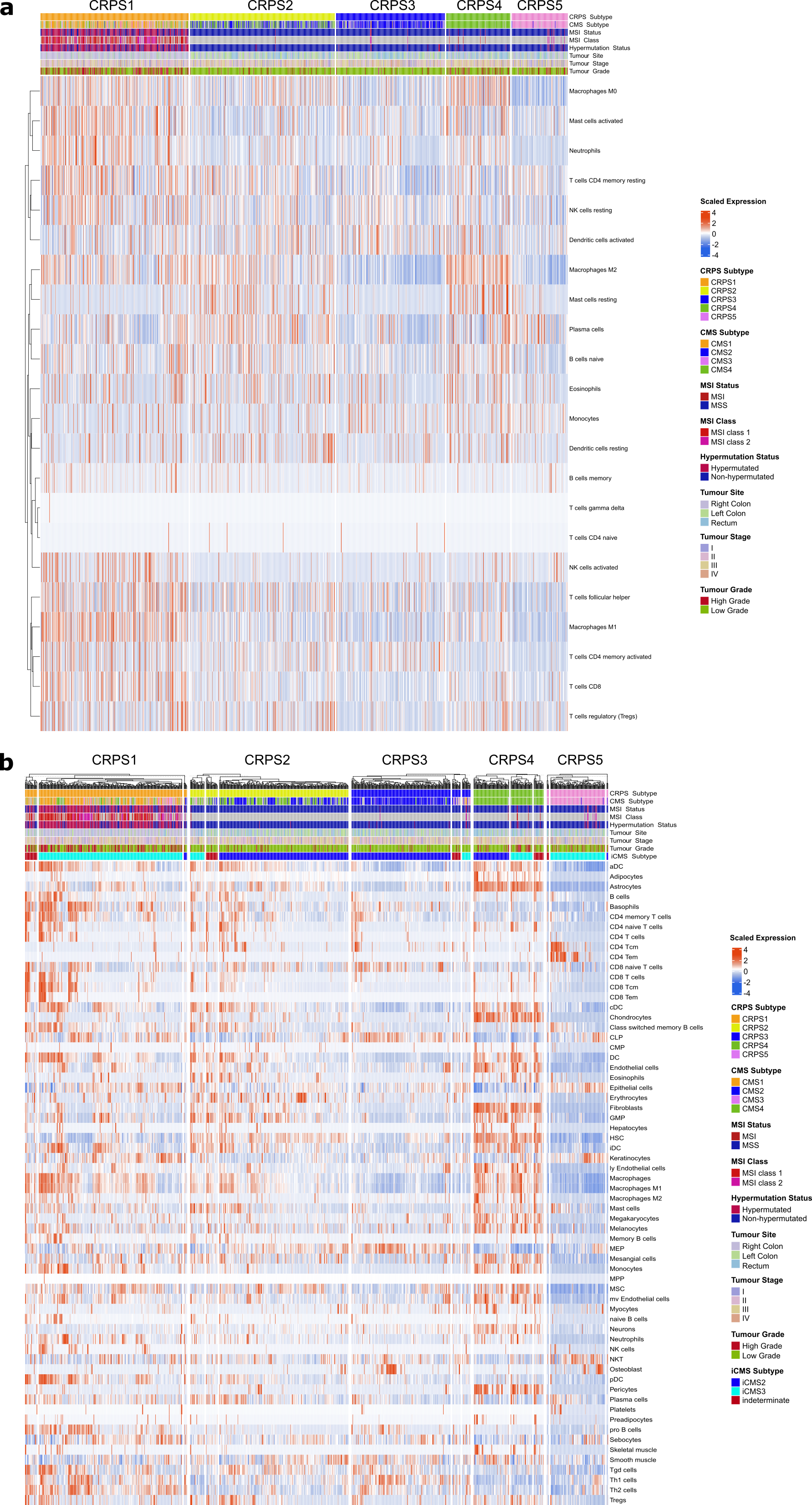


**Supplementary Figure 10. Immune and stromal cell composition of CRPSs**. Immune and

**Supplementary Figure 10 (continued).** stromal cells were predicted for each CRPS subtype using gene expression data and the (**a**) CIBERSORT and (**b**) xCell algorithms. Each row represents a cell and tumours were grouped by CRPS subtype. Clinical and genomic features are shown above each heatmap according to the respective colour schemes. Intrinsic subtypes of CMS (iCMS) are also indicated above the heatmap in **b**.


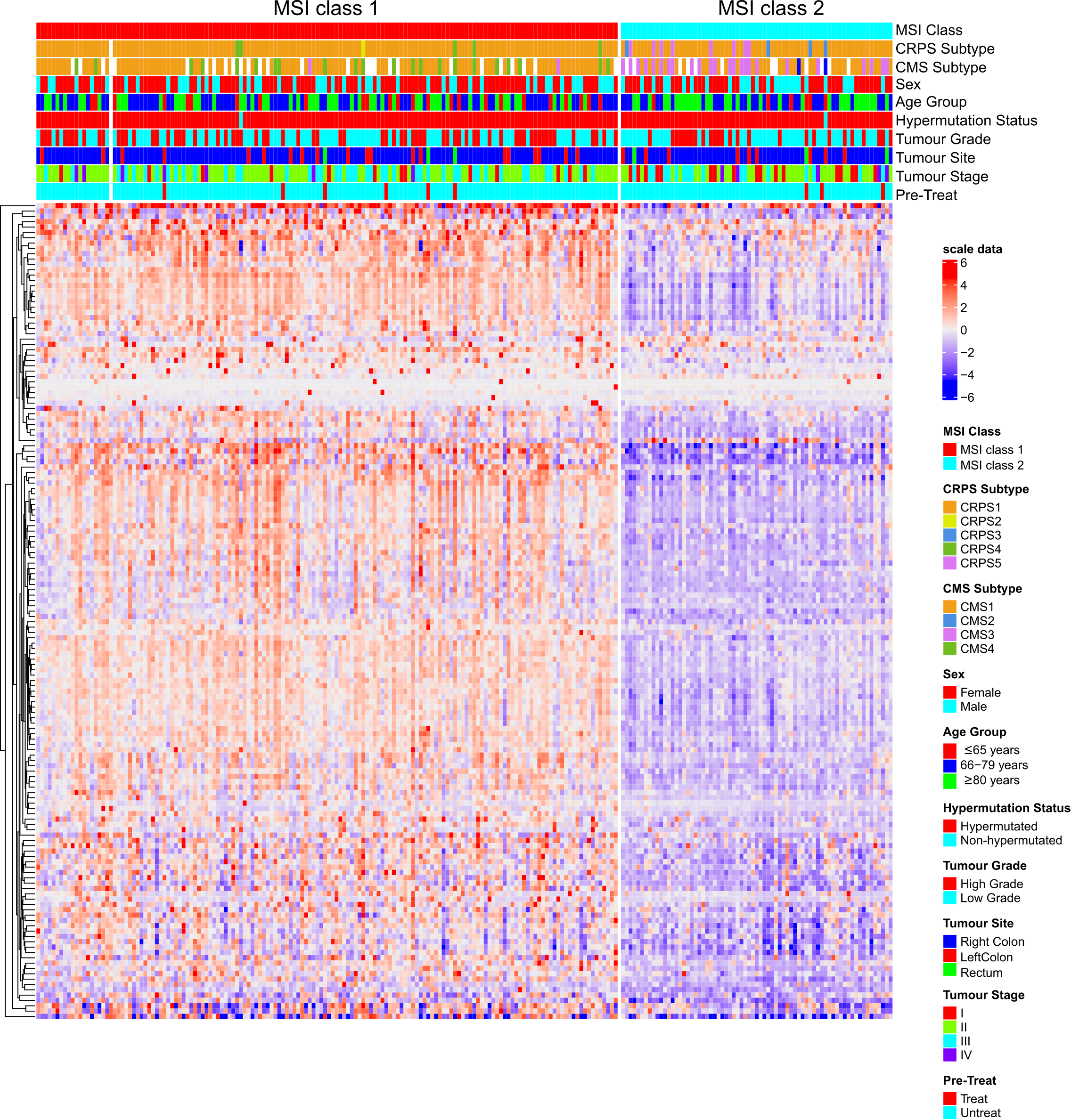


**Supplementary Figure 11. Unsupervised clustering reveals two subclasses of microsatellite instable CRCs.** Unsupervised clustering of transcriptome data divided microsatellite instable (MSI) colorectal cancer samples in two classes defined as class 1 (left) and class 2 (right). Heatmap of the top 150 marker genes in each MSI class, with red colour indicating high expression and blue colour indicating low expression of a particular gene. Other clinical and genomic features are represented according to respective colour schemes.


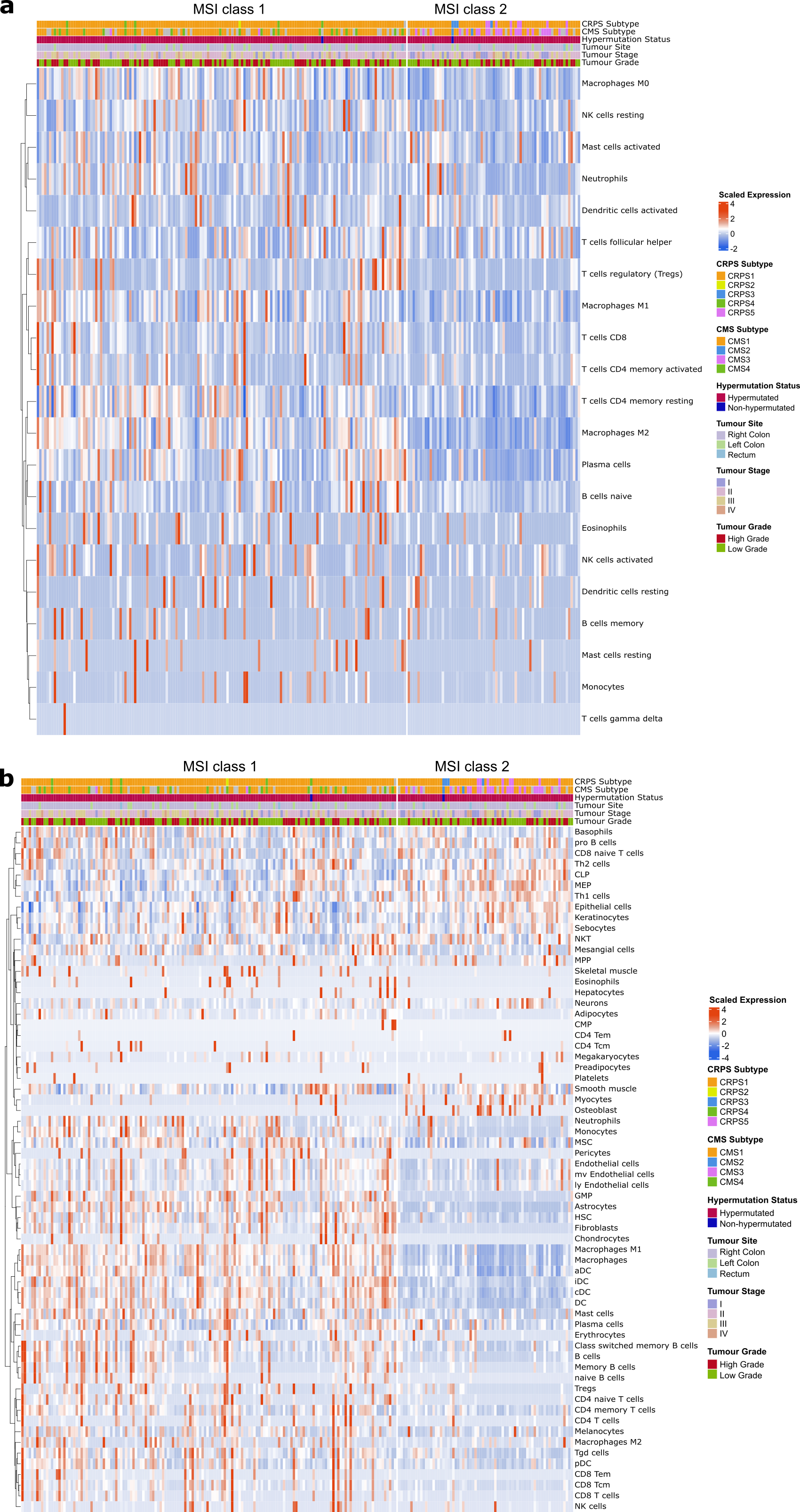


**Supplementary Figure 12. Infiltration of immune and stromal cell populations in**

**Supplementary Figure 12 (continued). microsatellite instable tumours**. Heatmaps of predictions of immune and stromal cell population for the two microsatellite instable (MSI) classes identified in this study for (**a**) CIBERSORT and (**b**) xCell algorithms. Each row represents a cell and tumours were grouped by MSI class. Clinical and genomic features represented above each heatmap according to respective colour schemes. MSI class 1 samples had higher frequency of infiltrated lymphocytes and stromal cells compared to MSI class 2 samples.

| **Supplementary Table 5. Patient and tumour characteristics by tumour hypermutation status.** | | | | |  |
| --- | --- | --- | --- | --- | --- |
| **Characteristics** | **All subjects**  **n = 1,063 (100%)** | **Non-Hypermutated**  **n = 821 (77%)** | **Hypermutated**  **n = 242 (23%)** | ***P*-value^#^** | |
| **Age (years)** |  |  |  |  | |
| Mean ± S.D. | 71.2 ± 11.3 | 70.3 ± 11.1 | 74.3 ± 11.3 |  | |
| Median (Range) | 72 (28-94) | 71 (28-94) | 76 (30-94) |  | |
| >75 years | 422 (40%) | 293 (36%) | 129 (53%) | **8.65e-6** | |
| **Sex** |  |  |  |  | |
| Female | 514 (48%) | 356 (43%) | 158 (65%) | **2.35e-9** | |
| **Primary Tumour Location** |  |  |  |  | |
| Right Colon | 498 (47%) | 301 (37%) | 197 (81%) | **<2.2e-16** | |
| Left Colon | 284 (27%) | 249 (30%) | 35 (15%) |  | |
| Rectum | 281 (26%) | 271 (33%) | 10 (4%) |  | |
| **Histology Subtype** |  |  |  |  | |
| Adenocarcinoma | 877 (83%) | 721 (88%) | 156 (64%) | **3.39e-15** | |
| Mucinous adenocarcinoma | 186 (17%) | 100 (12%) | 86 (36%) |  | |
| **Tumour Grade** |  |  |  |  | |
| Low grade | 837 (79%) | 700 (85%) | 137 (57%) | **<2.2e-16** | |
| **Tumour Stage** |  |  |  |  | |
| Stage I | 138 (13%) | 106 (13%) | 32 (13%) | **1.09e-9** | |
| Stage II | 392 (37%) | 261 (32%) | 131 (54%) |  | |
| Stage III | 419 (39%) | 354 (43%) | 65 (27%) |  | |
| Stage IV | 114 (11%) | 100 (12%) | 14 (6%) |  | |
| **Mismatch Repair Status** |  |  |  |  | |
| MSI | 223 (21%) | 2 (0%) | 221 (91%) | **<2.2e-16** | |
| MSS | 840 (79%) | 819 (100%) | 21 (9%) |  | |
| **Tumour Resection Surgery** |  |  |  |  | |
| Yes | 1034 (97%) | 799 (97%) | 240 (99%) | 0.136 | |
| **Pre-Treated** |  |  |  |  | |
| Radiation | 76 (7%) | 73 (9%) | 3 (1%) | **6.53e-5** | |
| Chemotherapy | 33 (3%) | 26 (3%) | 7 (3%) |  | |
| Chemoradiotherapy | 17 (2%) | 13 (2%) | 4 (2%) |  | |
| None | 938 (88%) | 709 (86%) | 228 (94%) |  | |
| **Survival** |  |  |  |  | |
| Median OS (95% CI) | 119 m (109-135) | 119 m (109–136) | 125 m (92–173) | 0.181 | |
| 5-Year OS Rate Stages I-III % | 75 % | 74 % | 77 % |  | |
| 5-Year OS Rate Stage IV % | 25 % | 26 % | 21 % |  | |
| Median RFS (95% CI)* | 121 m (111-136) | 121 m (113-136) | 96 m (82-173) | 0.109 | |
| 5-Year RFS Rate Stages I-III % | 69 % | 68 % | 74 % |  | |

**Supplementary Tables (non-Excel tables):**

MSI, microsatellite instable; MSS, microsatellite stable; OS, overall survival; RFS, recurrence free survival; CI, confidence interval. *RFS was calculated for stage I-III; ^#^*P*-value from two-sided Fisher’s exact test and log-rank test for survival comparisons between non-hypermutated and hypermutated cases, statistically significant (*P*<0.05) differences in bold.

| **Supplementary Table 6. Cohort characteristics compared to surgically treated colorectal cancer patients in Sweden 2007-2016.** | | | |
| --- | --- | --- | --- |
| ***Characteristics*** | **Present Cohort**  **n =1,063** | **Resected Patients in Sweden 2007-2016**  **n = 48,470** | ***P*-value^#^** |
| ***Age (years)*** |  |  |  |
| *>75 years* | 422 (40) | 18,751 (39) | 0.504 |
| ***Sex*** |  |  |  |
| *Female* | 514 (48) | 23,091 (48) | 0.664 |
| *Male* | 549 (52) | 25,379 (52) |  |
| ***Primary Tumour Location*** |  |  |  |
| *Colon* | 782 (74) | 33,957 (70) | **0.01** |
| *Rectum* | 281 (26) | 14,513 (30) |  |
| ***Colon Side Classification*** |  |  |  |
| *Right Colon* | 498 (64) | 18,754 (56) | **1.2e-5** |
| *Left Colon* | 284 (36) | 14,841 (44) |  |
| ***Tumour Stage*** |  |  |  |
| *Stage I* | 138 (13) | 9,103 (19) | **1.9e-7** |
| *Stage II* | 392 (37) | 16,404 (34) |  |
| *Stage III* | 419 (39) | 16,742 (34) |  |
| *Stage IV* | 114 (11) | 6,221 (13) |  |
| ***Surgery*** |  |  |  |
| *Tumour surgically removed* | 1,034 (97) | 48,470 (100) | - |
| *Not operated* | 29 (3) | - |  |
| ***5-year overall survival (OS)*** |  |  |  |
| *All Patients %* | 70% | 67% | - |
| *Stage I-III %* | 75% | 72% | - |
| *Stage IV %* | 25% | 28% | - |

Data from the Swedish Regional Cancer Center (RCC) quality register interactive report of the Swedish Colorectal Cancer Register (SCRCR) for colon and rectal cancers. ^#^*P*-value from two-sided Fisher’s exact test, statistically significant (*P*<0.05) differences in bold.

| **Supplementary Table 17. Clinical characteristics of 114 colorectal cancer patients with synchronous metastases.** | | | | |
| --- | --- | --- | --- | --- |
| ***Characteristics**** | **Right Colon**  **n = 43 (38%)** | **Left Colon**  **n = 36 (31%)** | **Rectum**  **n = 35 (31%)** | ***P*-value^#^** |
| ***Age (years)*** |  |  |  |  |
| *Mean ± S.D.* | 70.6 ± 11.5 | 67.6 ± 10.7 | 71.9 ± 8.5 |  |
| *Median (Range)* | 70 (44-89) | 68.5 (42-86) | 73 (56-88) |  |
| *>75 years* | 17 (40) | 9 (25) | 12 (34) | 0.405 |
| ***Sex*** |  |  |  |  |
| *Female* | 27 (63) | 17 (47) | 14 (40) | 0.114 |
| ***Histology Subtype*** |  |  |  |  |
| *Adenocarcinoma* | 30 (70) | 29 (81) | 32 (91) | 0.060 |
| *Mucinous adenocarcinoma* | 13 (30) | 7 (19) | 3 (9) |  |
| ***Tumour Grade*** |  |  |  |  |
| *Low grade malignancy* | 20 (47) | 30 (83) | 28 (80) | **0.0005** |
| ***Pre-Treated*** |  |  |  |  |
| *Yes* | 7 (16) | 7 (19) | 2 (6) | 0.191 |
| ***Metastases Location*** |  |  |  |  |
| *Liver* | 33 (77) | 27 (75) | 30 (86) | 0.524 |
| *Lung* | 10 (23) | 11 (31) | 19 (54) | **0.016** |
| *Peritoneum* | 13 (30) | 8 (22) | 3 (9) | 0.055 |
| *Lymph Node* | 5 (12) | 3 (8) | 7 (20) | 0.345 |
| *Bone* | 1 (2) | 1 (3) | 3 (9) | 0.444 |
| *Other* | 5 (12) | 1 (3) | 1 (3) | 0.221 |
| ***Treatment*** |  |  |  |  |
| *Best Supportive Care (BSC)* | 11 (26) | 5 (14) | 9 (26) | 0.381 |
| *Metastasectomy* | 21 (49) | 18 (50) | 10 (29) | 0.119 |
| *1^st^ Line chemotherapy* | 21 (49) | 29 (81) | 22 (63) | **0.014** |
| *2^nd^ Line* | 15 (35) | 14 (39) | 13 (37) | 0.969 |
| *3^rd^ Line ^+^* | 10 (23) | 6 (17) | 4 (11) | 0.414 |
| ***Median overall survival*** *(95% CI)* |  |  |  |  |
| *All* | 18 m (14-29) | 32 m (27-55) | 28 m (16-31) | 0.289 |
| *BSC only* | 10 m (1-18) | 21 m (7-41) | 10 m (7-34) | 0.239 |
| *Treated with metastasectomy* | 65 m (28-n/a) | 59 m (30-n/a) | 64 m (21-n/a) | 0.820 |
| *Treated without metastasectomy* | 15 m (7-20) | 31 m (20-46) | 28 m (11-30) | **0.0003** |

* Best Supportive Care (BSC) means that the patient did not receive metastasis surgery or tumour controlling systemic chemotherapy, whereas palliative radiotherapy could be provided. Most patients having metastasectomy also received peri-operative chemotherapy. ^#^*P*-value from two-sided Fisher’s exact test and log-rank test for survival comparisons, statistically significant (*P*<0.05) differences in bold.

**Supplementary Tables Captions:**

**Supplementary Table 1.** Clinical and sequencing data information for the 1,063 patients included in the study.

**Supplementary Table 2.** Two-way ANOVA test for DNA normal specimen types adjusted by multiple factors (tumour site, sex, age group, and tumour stage) in different analyses types.

**Supplementary Table 3.** Driver gene mutation and expression.

**Supplementary Table 4.** Hypermutation status based on tumour whole-genome sequencing mutational data.

**Supplementary Table 5.** Patient and tumour characteristics by tumour hypermutation status.

**Supplementary Table 6.** Cohort characteristics compared to surgically treat colorectal cancer patients in Sweden 2007-2016.

**Supplementary Table 7.** Mutual exclusivity and co-occurrence analysis for driver gene mutations by hypermutation status and tumour subtype.

**Supplementary Table 8.** Copy number variation for the autosomal driver genes by hypermutation status.

**Supplementary Table 9.** Evolutionary timing analyses by subgroups of non-hypermutated tumours.

**Supplementary Table 10.** Solution selection statistics for *de novo* single-base substitution, doublet-base substitution and small insertions and deletions mutational signatures.

**Supplementary Table 11.** De novo mutational signatures constitution and standard error of the mean by mutation type.

**Supplementary Table 12.** Cosmic decomposed *de novo* mutational signatures.

**Supplementary Table 13.** Activities of de novo and Cosmic decomposed mutational signatures by patient tumour.

**Supplementary Table 14.** Correlation analyses of mutational signatures.

**Supplementary Table 15.** Correlation of de novo single and doublet-base mutational signatures with mutational signatures found in Degasperi et al. 2022.

**Supplementary Table 16.** Mutations in the mitochondrial genomes of 1,063 colorectal cancers.

**Supplementary Table 17.** Clinical characteristics of 114 colorectal cancer patients with synchronous metastases.

**Supplementary Table 18.** Prognostic driver gene mutations.

**Supplementary Table 19.** Survival analyses of mitochondrial DNA mutations.

**Supplementary Table 20.** Expression analyses of coding genes linked to cCREs between samples with and without mutated non-coding elements.

**Supplementary Table 21.** Survival analyses of mutations in non-coding elements.

**Supplementary Table 22.** Survival analysis of cytoband CNVs by CMS, iCMS and CRPS subtype.

**Supplementary Table 23.** Survival analyses of structural variants by hypermutation status and CMS, iCMS and CRPS subtype.

**Supplementary Table 24.** Somatic alterations and gene expression levels in colorectal cancer signalling pathways.

**Supplementary Table 25.** Mean overall expression of driver genes in normal and tumour specimens by tumour sample mutation status.

**Supplementary Table 26.** Pathways, metabolism and immune signatures by CRPS group.

**Supplementary Table 27.** Summary of prognostic alterations linked to shorter or longer overall (OS) or recurrence-free (RFS) survival in non-hypermutated tumours by CRPS, CMS and iCMS subtype.

**Supplementary Table 28.** Correlation analyses between Buffa hypoxia scores and mutational features in all, non-hypermutated and hypermutated groups.

**Supplementary Table 29.** Immune cell predictions by CIBERSORT and xCELL.

**Supplementary Table 30.** Survival analyses by hypermutation, MSI or subtype for CIBERSORT and xCell predicted cell types.
