# Supplementary material for: Prognostic genome and transcriptome signatures in colorectal cancers
Source: Nature. 2024 Aug 7;633(8028):137–46. doi: 10.1038/s41586-024-07769-3 (PMC11374687; doi:10.1038/s41586-024-07769-3)
Supplement: Supplementary file 2 — Reporting Summary [file 41586_2024_7769_MOESM2_ESM.pdf]

nature portfolio

Corresponding author(s): Tobias Sjöblom

Last updated by author(s): May 29, 2024

Reporting Summary

Nature Portfolio wishes to improve the reproducibility of the work that we publish. This form provides structure for consistency and transparency in reporting. For further information on Nature Portfolio policies, see our Editorial Policies and the Editorial Policy Checklist.

Statistics

For all statistical analyses, confirm that the following items are present in the figure legend, table legend, main text, or Methods section.

n/a	Confirmed	
☐	☒	The exact sample size (*n*) for each experimental group/condition, given as a discrete number and unit of measurement
☐	☒	A statement on whether measurements were taken from distinct samples or whether the same sample was measured repeatedly
☐	☒	The statistical test(s) used AND whether they are one- or two-sided<br>*Only common tests should be described solely by name; describe more complex techniques in the Methods section.*
☐	☒	A description of all covariates tested
☐	☒	A description of any assumptions or corrections, such as tests of normality and adjustment for multiple comparisons
☐	☒	A full description of the statistical parameters including central tendency (e.g. means) or other basic estimates (e.g. regression coefficient) AND variation (e.g. standard deviation) or associated estimates of uncertainty (e.g. confidence intervals)
☐	☒	For null hypothesis testing, the test statistic (e.g. *F*, *t*, *r*) with confidence intervals, effect sizes, degrees of freedom and *P* value noted<br>*Give P values as exact values whenever suitable.*
☒	☐	For Bayesian analysis, information on the choice of priors and Markov chain Monte Carlo settings
☒	☐	For hierarchical and complex designs, identification of the appropriate level for tests and full reporting of outcomes
☐	☒	Estimates of effect sizes (e.g. Cohen's *d*, Pearson's *r*), indicating how they were calculated

Our web collection on statistics for biologists contains articles on many of the points above.

Software and code

Policy information about availability of computer code

Data collection	Data and metadata was extracted from the national quality registry, the Swedish Colorectal Cancer Registry (SCRCR), and completed from medical records, and no specific software was used.
Data analysis	Individual software components used in this study are as follows: SOAPnuke v2.0.7; Sentieon Genomics software v202010; Personal Cancer Genome Reporter (PCGR) v0.9.1; BRASS v6.3.4; ascatNgs v4.5; VAGrENT v3.7.0; facetsSuite v2.0.8; singularity v3.2.0; PrepareAA (git commit ID ba747ce); AmpliconClassifier v0.4.4; CINSignatureQuantification v1.0.0; MSIsensor2 v0.1 (git commit ID e0798c7); dNdScv v0.1.0 (git commit ID dcbf8e5); Maftools v2.12.0; GISTIC2.0 v2.0.23; SigProfilerExtraction v1.1.4; R v4.1.0 and v4.2.0; ActiveDriverWGS v1.1.2 (git commit ID 351ca77); GATK4 v4.2.0.0; pysam v0.15.3; PhylogicNDT v1.0 (git commit ID 84d3dd2); Bowtie2 v2.3.4.1; RNASeQC v2.3.6; STAR-Fusion v1.10.0; Arriba v2.1.0; STAR v2.7.1a and v2.7.8a; FusionAnnotator v0.2.0; annoFuse v0.91.0; Seurat v4.1.0; Celligner v1.0.1; scclusteval v0.0.09000; CMSclassifier v1.0.0; CMScaller v0.9.2; GSVA v1.42.0; scikit-rebate v0.62; Tensorflow v2.3.1; Imbalanced-learn v0.9.0; GSEA v4.2.3; PROGENy v1.16.0; PathwayMapper v2.3.0; Newt v3.0.5; DHARMa v0.4.5; CIBERSORT v1.04; xCell v1.1.0; R packages: stats v4.1.0, survival v3.3-1, survminer v0.4.9, finalfit v1.0.4. The CRPS clustering model (U-CAN_CRPS_Model v1.0.1) is available to use on https://github.com/SkymayBlue/U-CAN_CRPS_Model.

For manuscripts utilizing custom algorithms or software that are central to the research but not yet described in published literature, software must be made available to editors and reviewers. We strongly encourage code deposition in a community repository (e.g. GitHub). See the Nature Portfolio guidelines for submitting code & software for further information.

Data

Policy information about availability of data

All manuscripts must include a data availability statement. This statement should provide the following information, where applicable:

- Accession codes, unique identifiers, or web links for publicly available datasets
- A description of any restrictions on data availability
- For clinical datasets or third party data, please ensure that the statement adheres to our policy

Short somatic variant calls, copy number variation and structural variants data can be accessed at the European Variation Archive123 with accession number PRJEB61514, expression profiles at the ArrayExpress124 with accession number E-MTAB-12862, or all data at CNGB Sequence Archive (CNSA)125 of China National GeneBank DataBase (CNGBdb)126 with accession number CNP0004160. The raw transcriptome data generated in this paper is available under controlled access via EGA with accession number EGAD50000000169. WGS raw data and more detailed clinical information are deposited at Uppsala University and inquires to access them should be directed to the corresponding author and U-CAN, a cancer biobank at Uppsala University (https://www.uu.se/forskning/u-can/). Access to raw data and clinical information is subject to Swedish legal regulations, GDPR, permission from the Swedish Ethical Review Authority, and U-CAN terms. All patients in U-CAN have explicitly consented to genomic data deposition in public repositories. However, to protect their integrity and fulfil requirements in an evolving legal landscape, we have opted for restricted access to genome and transcriptome sequence datasets. Access requests can be addressed to the corresponding author and will be responded within 2 weeks. The remaining data are available within the Article and Supplementary Information.

The human genome reference hg38 (containing all alternate contigs) files were downloaded from GATK resource bundle (ftp.broadinstitute.org/gsapubftp-anonymous/bundle/hg38). The basic gene annotation file (gencode.v35.basic.annotation.gtf.gz) was downloaded from GENCODE (ftp.ebi.ac.uk/pub/databases/gencode/Gencode_human/release_35). A high-confidence list of genes with substantial published evidence in oncology (Cancer Gene Census v95) was downloaded from COSMIC (https://cancer.sanger.ac.uk/cosmic). A compendium of mutational cancer driver genes (release date 2020.02.01) was downloaded from intOGen (https://www.intogen.org/). COSMIC mutational signatures (version: v3.3) were downloaded from COSMIC (https://cancer.sanger.ac.uk/signatures/downloads/). Genomics England (GEL) 100,000 Genomes Project (100kGP) mutational signatures (science.abl9283_tables_s1_to_s33.xlsx) were downloaded from Science website (https://www.science.org/doi/10.1126/science.abl9283#supplementary-materials). The Registry of candidate cis-Regulatory Elements (cCREs V3) derived from ENCODE data were downloaded from SCREEN (https://screen.encodeproject.org/). Genome lib (GRCh38_gencode_v37_CTAT_lib_Mar012021.plug-n-play) used in STAR-Fusion was downloaded from CTAT genome lib (https://data.broadinstitute.org/Trinity/CTAT_RESOURCE_LIB/__genome_libs_StarFv1.10/). The Molecular Signatures Database (version: v7.4) was downloaded from MSigDB (https://www.gsea-msigdb.org/gsea/downloads.jsp).

Human research participants

Policy information about studies involving human research participants and Sex and Gender in Research.

Reporting on sex and gender	This study used the sex variable for the purpose of identifying and describing the study cohort and its results landscape. Additionally, the study findings were not specific to one sex, indicating that the results were applicable to both males and females. The sex variable was collected from the national quality registry based on patients' medical records. The cohort included 514 female sex (48%) and 549 male sex (52%) individuals. Patient consent and ethical permits were obtained for the use of this data.
Population characteristics	Detailed clinical data is provided in Supplementary Table 1, in line with other colorectal cancer population-based cohorts. Population ancestry is not registered in the clinical records and we didn't perform any ancestry-related analyses, however considering the geographical and demographical characteristics of this cohort, potentially most patients will be of European descent (more specifically Northern European).
Recruitment	Patients diagnosed with colorectal cancer between 2004 and 2019, at the Uppsala University Hospital or the Umeå University Hospital, were eligible for the study. Samples obtained had to meet criteria on sample availability and tumour cell content, meaning that the cohort represents patients that underwent surgery, leading to a small under-representation of stage I and IV cancers. There was no other major recruitment biases.
Ethics oversight	Patient inclusion, sampling and analyses were performed under the ethical permits 2004-M281, 2010-198, 2007-116, 2012-224, 2015-419, 2018-490 (Uppsala EPN), 2016-219 (Umeå EPN) and the Swedish Ethical Review Authority 2019-566. All participants provided written informed consent at enrolment. All samples were stored in the respective central biobank service facilities in Uppsala (Uppsala Biobank) and Umeå (Biobanken Norr) and obtained for use in analyses here after approved applications. Sequencing and sequence data analyses of pseudonymized samples were performed at BGI Research, which had access to patient age range, sex and tumour level data. Samples and data were transferred from UU to BGI Research under Biobank Sweden MTA and applicable GDPR standard terms for transfer to third countries. The analysis of patient-level data was performed at UU. The study conformed to the ethical principles for medical research involving human participants outlined in the Declaration of Helsinki.

Note that full information on the approval of the study protocol must also be provided in the manuscript.

Field-specific reporting

Please select the one below that is the best fit for your research. If you are not sure, read the appropriate sections before making your selection.

☒ Life sciences ☐ Behavioural & social sciences ☐ Ecological, evolutionary & environmental sciences

For a reference copy of the document with all sections, see nature.com/documents/nr-reporting-summary-flat.pdf

Life sciences study design

All studies must disclose on these points even when the disclosure is negative.

Sample size	Patients diagnosed with colorectal cancer between 2004 and 2019, at the Uppsala University Hospital or the Umeå University Hospital, were eligible for this study. Patients were included if they had i) a fresh frozen biopsy or surgical specimen that was estimated by a pathologist to have a tumour cell content of ≥20% and ii) a patient-matched source of normal DNA from whole blood or fresh frozen colorectal tissue stored in the biobank. Most samples were surgical specimens and treatment-naïve cases since these generally have enough tissue to be frozen besides the routine formalin-fixed paraffin-embedded storage. No statistical methods were used to predetermine sample size.
Data exclusions	Patients that had samples that fulfilled above criteria were excluded if at least one of the sample types (DNA tumour, DNA normal and RNA tumour) were not extracted with enough yield or quality, or if the respective sequencing were of inadequate coverage or evidence for cross-contamination between samples.
Replication	To validate the novel Colorectal Caner Progonstic Subtypes (CRPS) classification, we built a classification model based on the deep residual learning framework. To test our CRPS clustering model reproducibility, a total of 10 colorectal cancer data sets with 2,832 patients from both NCBI GEO and NCI Genomic Data Commons were uniformly processed. The accuracy, precision, recall and F1 score were >85% in the validation cases and the prognostic ability of CRPS was recapitulated in this external validation. Replication may not be applicable to the other landscape findings that are descriptive for this cohort. All attempts at replication were successful.
Randomization	No randomisation was performed - this was a descriptive study, not an experimental study.
Blinding	No blinding was undertaken - this was a descriptive study, not an experimental study.

Reporting for specific materials, systems and methods

We require information from authors about some types of materials, experimental systems and methods used in many studies. Here, indicate whether each material, system or method listed is relevant to your study. If you are not sure if a list item applies to your research, read the appropriate section before selecting a response.

Materials & experimental systems

n/a	Involved in the study	
☒	☐	Antibodies
☒	☐	Eukaryotic cell lines
☒	☐	Palaeontology and archaeology
☒	☐	Animals and other organisms
☐	☒	Clinical data
☒	☐	Dual use research of concern

Methods

n/a	Involved in the study	
☒	☐	ChIP-seq
☒	☐	Flow cytometry
☒	☐	MRI-based neuroimaging

Clinical data

Policy information about clinical studies

All manuscripts should comply with the ICMJE guidelines for publication of clinical research and a completed CONSORT checklist must be included with all submissions.

Clinical trial registration	Not Applicable
Study protocol	Patients diagnosed with CRC between 2004 and 2019, at Uppsala University Hospital or Umeå University Hospital, were eligible for the study. Patients that had i) a fresh frozen biopsy or surgical specimen that was estimated by a pathologist to have a tumour cell content of ≥20% and ii) a patient-matched source of normal DNA from whole blood or fresh frozen colorectal tissue stored in the biobank, were included. Patients included with a diagnosis from 2010 (861 cases; 81%) were obtained from the Uppsala-Umeå Comprehensive Cancer Consortium (U-CAN) biobank collections (Uppsala Biobank and Biobanken Norr). A description of procedures for patient inclusion, sample biobanking, and access to samples can be found at https://www.uu.se/forskning/u-can/.
Data collection	Clinical data was extracted from the national quality registry, the Swedish Colorectal Cancer Registry (SCRCR), and completed from medical records.
Outcomes	Follow-up for alive patients was minimum 3.9 years and median 8 years (data lock 14th June 2023), with only one patient lost to follow-up and 994 (94%) with complete 5-year follow up.

March 2021
